# From the Gut to the Brain: Modeling Intestinal Absorption and Assessing Blood–Brain-Barrier Permeability of Small-Molecule Drugs by IAM Chromatography

**DOI:** 10.1021/acsomega.6c00701

**Published:** 2026-06-25

**Authors:** Stefan Simić, Predrag Kalaba, Judith Wackerlig, Thierry Langer

**Affiliations:** † Department of Pharmaceutical Sciences, University of Vienna, Josef-Holaubek-Platz 2, 1090 Vienna, Austria; ‡ Vienna Doctoral School of Pharmaceutical, Nutritional and Sport Sciences, University of Vienna, Josef-Holaubek-Platz 2, 1090 Vienna, Austria

## Abstract

Designing small-molecule compounds and delivery systems capable of crossing the blood–brain barrier (BBB) remains a major obstacle in central nervous system (CNS) drug development. This challenge is compounded by the lack of robust, rapid, and cost-effective methods for early-stage assessment of BBB permeability. In this study, we addressed this gap by evaluating immobilized artificial membrane (IAM) chromatography as a high-throughput screening tool using a set of 50 marketed drugs with diverse chemical structures and well-documented CNS penetration profiles (both positive and negative). The strong correlation between IAM retention parameters (*k*_IAM_) and passive diffusion-based permeability confirms the ability of this biomimetic method to simulate drug–membrane interactions, establishing IAM chromatography as a reliable and economical alternative to more complex cell-based and *in vivo* models. Additionally, the integration of IAM data with *in silico* molecular descriptors yields a predictive model for intestinal absorption, offering a powerful tool for early permeability profiling in drug discovery.

## Graphical abstract

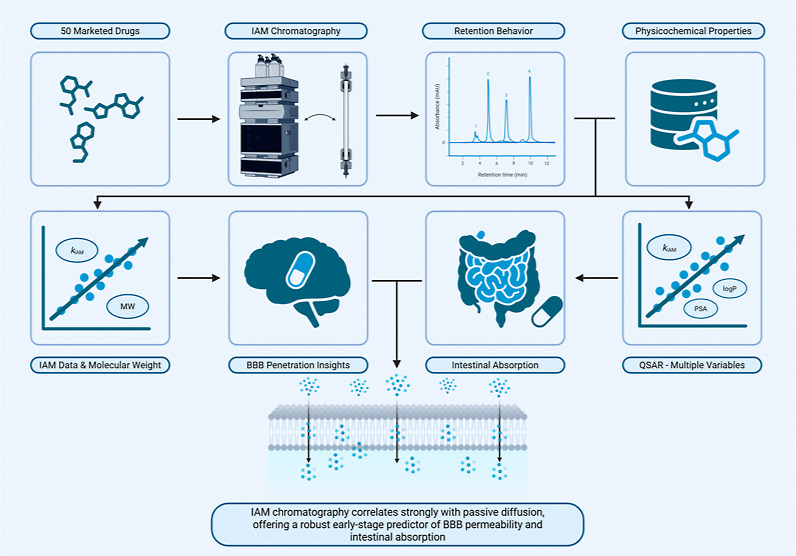

## 1 Introduction

The blood–brain barrier (BBB) serves as a highly selective interface between the bloodstream and the central nervous system (CNS), effectively shielding the brain from toxins, pathogens, and fluctuations in plasma composition.^1–5^ However, this very same protective function of the BBB significantly limits the capability of therapeutic compounds to reach the prospective CNS targets, creating a major challenge in drug discovery for both small-molecule drugs and larger biopharmaceuticals.^2,6^ So far, 98% of small-molecule drugs and virtually all large-molecule therapeutics are prevented from entering the brain.^2,6^ This is also reflected by the number and chemical nature of the novel therapeutics approved for the treatment of CNS diseases by the US Food and Drug Administration (FDA) between 2016 and 2020, with the majority of them being new small-molecule entities.^2^ Over the past decade, even small-molecule drugs had an approval rate that was approximately 40% lower compared to non-CNS drugs. This, together with the generally higher cost of drug discovery and development, has led to the abandonment of research in the CNS area by many companies. However, one has to keep in mind the large market opportunities and unmet medical needs for many CNS-related diseases.^7^

Given these challenges, modulating BBB permeability for selective delivery of therapeutics represents one of the top priorities in CNS drug discovery.^6–8^ To tackle them, it is worth emphasizing that drug penetration into the brain occurs primarily through passive diffusion, whereas large polar molecules require carrier-mediated transport or receptor-mediated endocytosis.^5,9–11^ Carrier-mediated and receptor-mediated mechanisms, combined with recent advances in material sciences and nanotechnology, have led to the development of various strategies for regulation of BBB permeability.^2,5,6^ Drugs that can cross the BBB via passive transport are limited to liposoluble compounds with a molecular weight lower than 400–600 Da.^5,12,13^ Hence, increasing the lipophilicity of therapeutic agents has traditionally been a feasible strategy to improve the BBB permeability, although other approaches such as reducing hydrogen bond donor capacity, reducing total polar surface area (tPSA), rigidity enhancement, and p*K*_a_ reduction, have been used as important parameters in drug design in the recent years.^2,14–17^

Extensive efforts by medicinal chemists have led to updating the repertoire of structural modifications to influence BBB penetration through modulation of either passive diffusion or active transport.^2^ To assess the BBB penetration potential of drugs and optimize CNS drug delivery in early drug development, numerous methods are used: computational models, *in vitro* BBB models (e.g., endothelial cell monolayers), and *in vivo* imaging techniques such as positron emission tomography (PET).^5,10^ Compared to *in silico* models, *in vitro* methods offer greater accuracy, and compared to *in vivo* studies, they are more efficient and less labor-intensive. As such, they provide fast, reliable, and cost-effective platforms for high-throughput screening, commonly used to predict BBB penetration and intestinal absorption to evaluate potential drug candidates.^2,5,11,18–24^

Recent advances in microfluidic BBB-on-chip and organ-on-chip platforms have substantially improved the physiological relevance of *in vitro* BBB models by introducing dynamic perfusion, controlled shear stress, three-dimensional microenvironments, and multicellular neurovascular-unit architectures. In contrast to conventional static Transwell-based systems, these platforms can better reproduce key BBB characteristics such as tight-junction organization, endothelial polarization, transporter activity, barrier maturation, and regulated molecular permeability under flow conditions.^25–27^ Reviews of recent BBB-on-chip technologies emphasize that the integration of human brain endothelial cells with astrocytes, pericytes, neurons, microglia, and extracellular matrix components allows these models to more closely reflect structural, cellular, and biochemical complexity of the human BBB.^28–31^ These advances are particularly relevant for CNS drug development, where insufficiently predictive static *in vitro* models may contribute to poor translation of permeability and therapeutic efficacy.^29,30^

Several representative studies demonstrate the practical value of microfluidic BBB models for drug permeability testing and physiological barrier assessments. Wang et al. developed a microfluidic BBB model that displayed more *in vivo*-like barrier properties and was suitable for drug permeability screening, illustrating the potential of flow-based systems to improve prediction of compound transport across the BBB.^26^ Hajal et al. provided a detailed protocol for an engineered human microfluidic BBB model designed for vascular permeability analysis, thus further supporting the use of standardized microfluidic platforms for quantitative barrier function studies.^27^ More recently, Yang et al. used a microfluidic chip to evaluate BBB permeability of multiple drugs, demonstrating the application of these systems for early CNS permeability assessment under more physiologically relevant conditions than static culture models.^32^ Together, these studies support the view that microfluidic BBB platforms can provide more dynamic and predictive readouts of barrier function, especially when permeability, endothelial response, and transport behavior are central experimental end points.^26,27,32^

Beyond permeability testing, newer BBB-on-chip and brain-chip systems increasingly incorporate advanced readouts and disease- or therapy-oriented applications. Ceccarelli et al. developed a sensorized three-dimensional BBB-on-chip platform that enabled real-time monitoring of barrier maturation and integrity, highlighting the value of integrated sensing technologies for noninvasive assessment of BBB function over time.^33^ Choi et al. applied an organ-on-chip approach to accelerate the discovery of BBB-crossing nanoshuttles, showing how these platforms can support screening of brain-targeted delivery systems under conditions that better approximate the *in vivo* BBB microenvironment.^34^ Chim et al. further demonstrated the utility of a human brain-chip platform for modeling brain pathologies and evaluating therapeutic strategies capable of crossing the BBB, expanding the relevance of organ-on-chip approaches beyond permeability assays alone.^35^ Although these technologies offer clear advantages, broader implementation still requires improved standardization of cell sources, chip geometries, flow conditions, end point measurements, and validation against human clinical or *in vivo* data.^29,31,36^ Overall, BBB-on-chip and organ-on-chip systems represent promising next-generation tools that complement conventional BBB models and may improve the translational relevance of CNS drug development, neurotoxicity testing, and disease modeling.^28,30,31^

Furthermore, *in vivo* imaging approaches, including positron emission tomography (PET), enable direct assessment of brain exposure and pharmacokinetics in living organisms.^37^ In recent years, quantitative pharmacokinetic metrics such as the unbound brain-to-plasma partition coefficient (*K*_p,uu_) have become increasingly important for characterizing the contribution of active transport and efflux mechanisms at the BBB.^38^

One of the methods that has recently emerged is Immobilized Artificial Membrane (IAM) chromatography, a specialized chromatographic technique used to assess drug–membrane interactions. It is based on phospholipid-immobilized stationary phases, where phosphatidylcholine chains are covalently bound to silica particles, mimicking biological membrane interactions more effectively than traditional reversed-phase high-performance liquid chromatography. This allows for the accurate simulation of passive diffusion processes, enabling the assessment of membrane permeability in a physiologically relevant context.^39–45^

The aim of this study was to evaluate IAM chromatography as a predictive tool for both intestinal absorption and BBB penetration, using a data set of structurally diverse, therapeutically approved and marketed compounds. By comparing data obtained through IAM experiments with quantitative structure–activity relationship (QSAR) modeling, we investigate how common physicochemical drug properties such as logarithm of the partition coefficient (logP), polar surface area (PSA), and molecular weight (MW) influence passive permeability. The findings will provide insights into the applicability of IAM chromatography in drug discovery, particularly in early-stage screening for CNS-active drugs and oral bioavailability assessments.

## 2 Materials and Methods

### 2.1 Test Compounds

All tested compounds, as well as chemicals used for buffer preparation, were purchased from Sigma-Aldrich (Schnelldorf, Germany), Gall Pharma (Judenburg, Austria), and TCI-Europe (Zwijndrecht, Belgium) as pharmaceutical standards in the highest available purity. Solvents used in chromatography were obtained from Merck (Darmstadt, Germany) in HPLC and LC–MS grade. Dulbecco’s phosphate-buffered saline (DPBS), used for mobile phase, was purchased from Sigma-Aldrich (Schnelldorf, Germany).

Stock solutions of the standards used in the biomimetic chromatography evaluation were prepared at a concentration of 1 mg/mL in DMSO. Samples were prepared by mixing the stock solution with Dulbecco’s phosphate-buffered saline containing 20% acetonitrile (1:1, v/v) to obtain final concentrations of 0.5 mg/mL.

### 2.2 HPLC Conditions

Experiments were conducted using a Nexera XR UHPLC system (Shimadzu Corporation, Tokyo, Japan) equipped with an IAM.PC.DD.2 column (100 × 4.6 mm, 10 μm; Regis Technologies, Morton Grove, USA). Chromatographic conditions included a flow rate of 1.0 mL/min, with the column temperature maintained at 37 °C to simulate body temperature. The mobile phase for the blood–brain barrier studies consisted of 20% acetonitrile and 80% Dulbecco’s phosphate-buffered saline (DPBS) at pH 7.0 ± 0.05, adjusted with sodium hydroxide. For intestinal absorption screening, the pH of DPBS was adjusted to 5.5 ± 0.05 using 2 M hydrochloric acid. The DPBS buffer contained 2.7 mM KCl, 1.5 mM KH_2_PO_4_, 137 mM NaCl, and 8.1 mM Na_2_HPO_4_. Acetonitrile was added as an organic modifier to the mobile phase to reduce analyte retention times by decreasing the polarity of the eluent, thereby increasing elution strength. The injection volume was 2 μL, and the diode array detector was set to detect wavelengths between 190 and 400 nm.

### 2.3 IAM Chromatography: Coefficient of Partition in the BBB and Intestinal Absorption Study

For the determination of the IAM partition coefficients for the BBB assay, each measurement was conducted in triplicate. The IAM retention (capacity) factor (k_IAM_) (Table 1) was determined using the following equation

1 $$k_{IAM}=\frac{t_{R}-t_{0}}{t_{0}}$$

In eq 1, *t*_R_ represents the retention time of the compound and *t*_0_ corresponds to the void volume (determined by injecting uracil with the sample).

**Table 1:** Summary of Parameters used for Calculations and their Physical Meaning

symbol	definition	physical meaning
*k*_IAM_	IAM retention (capacity) factor	chromatographic retention factor describing the interaction of a compound with the IAM stationary phase
*K*_IAM_	IAM equilibrium partition coefficient	partitioning of the compound between the IAM phospholipid layer and the mobile phase
*K*_m_	membrane partition coefficient	equilibrium partitioning of the compound between the lipid membrane of the IAM column and the mobile phase
*P*_m_	membrane permeability coefficient	rate at which a compound diffuses across a biological membrane
Β	phase ratio	ratio of the stationary phase interfacial volume to the mobile phase volume within the IAM column
*V*_m_	mobile phase volume	total solvent volume within the IAM column
*V*_s_	stationary phase interfacial volume	interphase volume created by immobilized phospholipids within the column
*D*_m_	diffusion coefficient in the membrane	diffusion rate of the compound within the lipid bilayer
*L*	membrane thickness	thickness of the lipid bilayer (approximately 30 Å)

The capacity factor (*k*_IAM_) shows a direct relation to the equilibrium IAM partition coefficient (*K*_IAM_) (Table 1) as follows

2 $$k_{IAM}=\frac{V_{s}}{V_{m}}K_{IAM}=\beta K_{IAM}$$

where *V*_m_ stands for the total solvent volume within the IAM column, *V*_S_ denotes the interfacial volume formed by the immobilized phospholipids, and β = *V*_s_/*V*_m_ (Table 1) is the phase ratio characteristic of the column. Since the exact IAM interfacial thickness remains unknown, accurately measuring *V*_S_ is challenging.^46^ The equilibrium drug partitioning into fluid membranes typically serves as a limiting factor in drug penetration. For compounds absorbed via passive diffusion, membrane permeability (*P*_m_) (Table 1) is directly proportional to the equilibrium membrane partition coefficient (*K*_m_) (Table 1)­

3 $$P_{m}=\frac{D_{m}K_{m}}{L}$$

where *D*_m_ is the diffusion coefficient of the drug within the membrane and *L* (Table 1) is the membrane thickness (approximately 30 Å for the hydrocarbon domain of bilayers). Given that the IAM system is designed to mimic fluid membranes, it is assumed that *K*_IAM_ and *K*_m_ exhibit a linear correlation.^47–49^

Since diffusion coefficients (*D*_m_) (Table 1) are influenced by molecular size, the following proportionality is applied

4 $$D_{m}\propto \frac{1}{V^{n}}$$

where *V* denotes the molar volume of the drug and η is a constant. Under the assumption that the molecular weight (MW) is proportional to the molar volume, *D*_m_.^48,49^ This can be approximated as

5 $$D_{m}\propto \frac{1}{{MW}^{n}}$$

As a result, the membrane permeability (*P*_m_) for passively diffusing drugs can be expressed as

6 $$P_{m}\propto \frac{k_{IAM}}{{MW}^{n}}$$

In the study of Yoon et al., after evaluating various power functions, it was determined that the best correlation between IAM capacity factors and blood–brain barrier (BBB) permeability occurred when the power function was set to *n* = 4.^48^ Thus, the most suitable expression for predicting passive permeability was found to be

7 $$P_{m}=\frac{k_{IAM}}{{MW}^{4}}\times 10^{10}$$

### 2.4 BBB and Human Intestinal Absorption Parameters

The data used in the study consists of literature-reported information on whether specific compounds can cross the blood–brain barrier, recorded as categorical outcomes (positive/negative). These data were gathered from multiple sources, including experimental studies and predictive models, to ensure a comprehensive and reliable analysis of BBB permeability. The examined compounds are known to cross the BBB primarily through passive diffusion.^48,50–61^

When the percentage of the human intestinal absorption (%HIA) values varied across sources or were presented as a range, the mean value was used. Alternatively, if a specific value was more frequently reported, it was selected as the representative value.^3,42,43,60,62–64^ For cefuroxime, the pro-drug form cefuroxime axetil was used. Cefuroxime has a rather poor intestinal absorption due to its hydrophilicity, but its prodrug is a lipophilic ester that increases its intestinal absorption.^65^

The physicochemical descriptors including topological polar surface area, logP, molecular weight, molecular volume (MV), molar refractivity (MR), and surface tension (ST) were calculated with the use of BIOVIA Dassault Systèmes, Draw 2022 (Dassault Systèmes, San Diego, USA).

### 2.5 Statistical Analysis

Receiver operating characteristic (ROC) analysis was performed to evaluate CNS permeability and the classification performance of the *P*_m_ descriptors. ROC curves and the corresponding area under the curve (AUC) were generated using MedCalc Statistical Software version 23.4.9 for Windows 11 (MedCalc Software, Ostend, Belgium), while complementary statistical analyses were conducted using IBM SPSS Statistics 30 for Windows 11 (IBM Corp, Armonk, USA). The optimal classification threshold was identified by using the Youden index.

IBM SPSS Statistics 30 for Microsoft Windows 11 (IBM Corp., Armonk, USA) was utilized to construct the QSAR model, employing multiple forward stepwise regression to identify the most relevant *k*_IAM_ and molecular descriptors for predicting intestinal absorption (%HIA) as the dependent variable. Initially, a model was created featuring all of the listed independent variables, which are known to influence intestinal absorption. These included the partition coefficient logP, polar surface area, molecular weight, molar volume, and surface tension. Afterward, the statistically not significant variables were removed (*p* > 0.005), and additional variables were sequentially created and added until no further improvement in the relationship was observed. The regression equations were evaluated by using the correlation coefficient (*R* ^2^), standard deviations (*s*), and the Fisher test (*F*) at a 95% significance level.

## 3 Results and Discussion

### 3.1 Blood–Brain Barrier Permeability of Tested Compounds

The data set of 50 compounds (Table 2) with literature-documented CNS-permeability exhibited clear distinctions between CNS-permeable (CNS+) and nonpermeable (CNS−) compounds when comparing their *P*_m_ values. As shown in Figure 1, compounds classified as CNS+ demonstrated *P*_m_ values above the established threshold of 1.51 (metoclopramide), while CNS– compounds fell below the value of 1.43 (pilocarpine).

**Table 2:** BBB Penetration Estimation Based upon Immobilized Artificial Membrane Chromatography^a^

compound	CNS	MW	*P*_m_	*k*_IAM_ [*n* = 3] ± CV [ %]
atenolol	–	266.3	0.62	0.31 ± 0.34
baclofen	–	213.7	0.83	0.17 ± 1.00
bisoprolol	–	325.4	0.01	0.01 ± 4.45
cefuroxime axetil	–	510.5	0.00	0.00 ± 0.22
chlorothiazide	–	295.7	0.05	0.04 ± 4.55
cimetidine	–	252.3	0.86	0.35 ± 0.59
domperidone	–	425.9	0.00	0.00 ± 0.36
enalapril	–	367.5	0.15	0.28 ± 0.76
etoposide	–	588.6	0.10	1.15 ± 0.15
glimepiride	–	452.6	0.14	0.78 ± 0.45
furosemide	–	330.7	0.31	0.37 ± 0.46
hydrochlorothiazide	–	297.7	0.42	0.33 ± 0.61
losartan	–	422.9	0.43	1.39 ± 0.12
miconazole	–	416.1	0.00	0.00 ± 0.28
nadolol	–	309.4	0.69	0.63 ± 0.62
nizatidine	–	331.5	0.25	0.30 ± 3.55
parecoxib	–	370.4	0.39	0.73 ± 0.66
pilocarpine	–	224.7	1.43	0.36 ± 0.37
prazosin	–	419.9	0.66	2.05 ± 0.21
ranitidine	–	350.9	0.29	0.44 ± 0.70
repaglinide	–	452.6	0.19	0.81 ± 0.85
sotalol	–	272.4	0.84	0.46 ± 0.29
theophylline	–	180.2	1.05	0.11 ± 0.52
timolol	–	316.4	1.31	1.31 ± 0.68
torasemide	–	348.4	0.46	0.68 ± 0.81
amiloride	+	229.6	5.91	1.64 ± 0.22
barbital	+	184.2	2.77	0.32 ± 0.24
caffeine	+	194.2	7.33	1.04 ± 0.50
carbamazepine	+	236.3	10.76	3.35 ± 0.18
clonidine	+	230.1	5.25	1.47 ± 0.07
desipramine	+	266.4	27.27	13.73 ± 0.61
diazepam	+	284.7	7.03	4.62 ± 1.33
diltiazem	+	414.5	2.30	6.80 ± 0.63
donepezil	+	416.0	2.20	6.60 ± 0.60
ethacridine	+	264.4	23.15	11.31 ± 0.25
ibuprofen	+	206.3	5.27	0.95 ± 0.41
melatonin	+	232.3	4.37	1.27 ± 3.96
metoclopramide	+	299.8	1.51	1.22 ± 0.95
modafinil	+	273.4	3.50	1.95 ± 1.37
noscapine	+	413.4	2.79	8.16 ± 0.87
oxazepam	+	286.7	7.29	4.93 ± 0.35
phenobarbital	+	232.2	3.38	0.98 ± 0.33
prednisolone	+	360.4	1.88	3.17 ± 0.01
propranolol	+	259.3	4.51	2.04 ± 0.98
quinidine	+	324.4	8.83	9.78 ± 0.12
rivastigmine	+	250.3	4.46	1.75 ± 0.15
salicylic acid	+	138.1	7.81	0.28 ± 1.72
scopolamine	+	303.4	1.57	1.33 ± 0.34
tetracaine	+	264.4	15.63	7.63 ± 2.58
zolpidem	+	307.3	2.83	2.52 ± 0.19

a Symbol definitions: MW, molecular weight; *P*_m_, coefficient of membrane permeability; *k*_IAM_, retention factor; CV, coefficient of variation.

**Figure 1:**
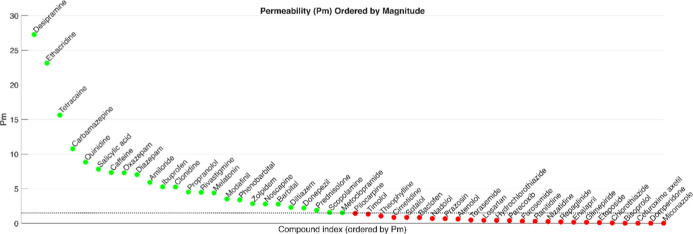
Classification of CNS– with the established *P*_m_ threshold of 1.43 and CNS+ drugs based on *P*_m_ at 1.51.

The IAM retention factors were determined in triplicate and are reported together with the coefficient of variation (CV). In chromatographic measurements of retention factors, CV values below approximately 5% are generally considered indicative of good analytical reproducibility, which is consistent with previously reported IAM chromatography studies.^40,41,43^ In the present data set, most compounds exhibited CV values well within this range, demonstrating the robustness of the experimental setup. Compounds with very low retention factors tend to exhibit slightly higher CV values, which is a known limitation in chromatographic measurements when the retention time approaches the void volume.

To quantitatively evaluate the ability of the *P*_m_ descriptor to discriminate between CNS-penetrant and nonpenetrant compounds, receiver operating characteristic (ROC) analysis was performed. ROC curves were generated by plotting the true positive rate (sensitivity) against the false positive rate (1 – specificity) across the full range of possible *P*_m_ thresholds. The area under the ROC curve (AUC) (Figure 2) was used as a global measure of classifier performance, with values approaching 1 indicating excellent discrimination between the two classes.

**Figure 2:**
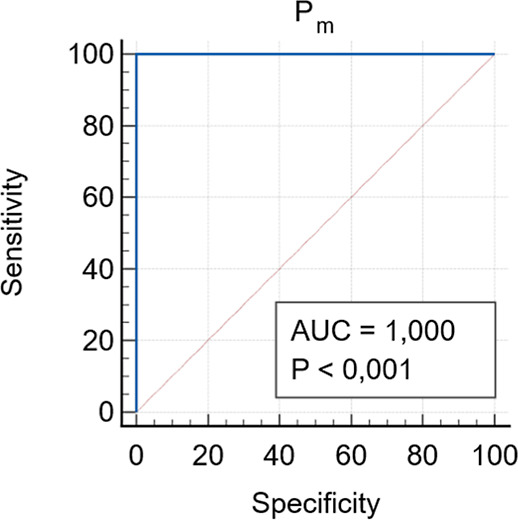
ROC curve for *P*_m_-based CNS classification.

The optimal classification threshold was determined using the Youden index (*J* = sensitivity + specificity – 1), which identifies the cutoff value that maximizes the combined sensitivity and specificity of a binary classifier. The optimal classification threshold was determined using the Youden index, yielding an optimal cutoff of *P*_m_ value of 1.51. At this threshold, the model achieved a sensitivity of 1.00, a specificity of 1.00, and a balanced accuracy of 1.00 (Table 3). The resulting confusion matrix (Table 4) showed correct classification of all CNS-positive and CNS-negative compounds.

**Table 3:** Statistical Performance Metrics for *P*_m_-Based Classification of CNS Penetration

metrics	value
optimal threshold (Youden index)	*P*_m_ ≈ 1.51
ROC AUC	1.00
sensitivity (true positive rate)	1.00
specificity (true negative rate)	1.00
accuracy	1.00
balanced accuracy	1.00
Matthews correlation coefficient	1.00

**Table 4:** Confusion Matrix for *P*_m_-Based Classification of CNS Penetration

	predicted CNS–	predicted CNS+
actual CNS–	25	0
actual CNS+	0	25

The Matthews correlation coefficient (MCC) was additionally calculated as a robust summary statistic derived from the confusion matrix that incorporates all four classification outcomes (true positives, true negatives, false positives, and false negatives). The MCC was 1.00, indicating perfect separation within the current data set. While the classification performance is excellent within the present data set, the relatively small sample size highlights the need for future validation using independent compound sets.

In general, the CNS drugs (Figure 3) showed the impact of polar functional groups and ionizable groups on the ability to pass the BBB. Compounds such as cefuroxime (*P*_m_ = 0.00) and furosemide (*P*_m_ = 0.31) illustrate the negative effect of multiple polar groups on BBB permeability.^16,17^ Cefuroxime axetil contains a carboxylic acid moiety and two amide groups, which are prone to hydrogen bonding and thus reduce its ability to pass through the lipophilic environment of the BBB.

**Figure 3:**
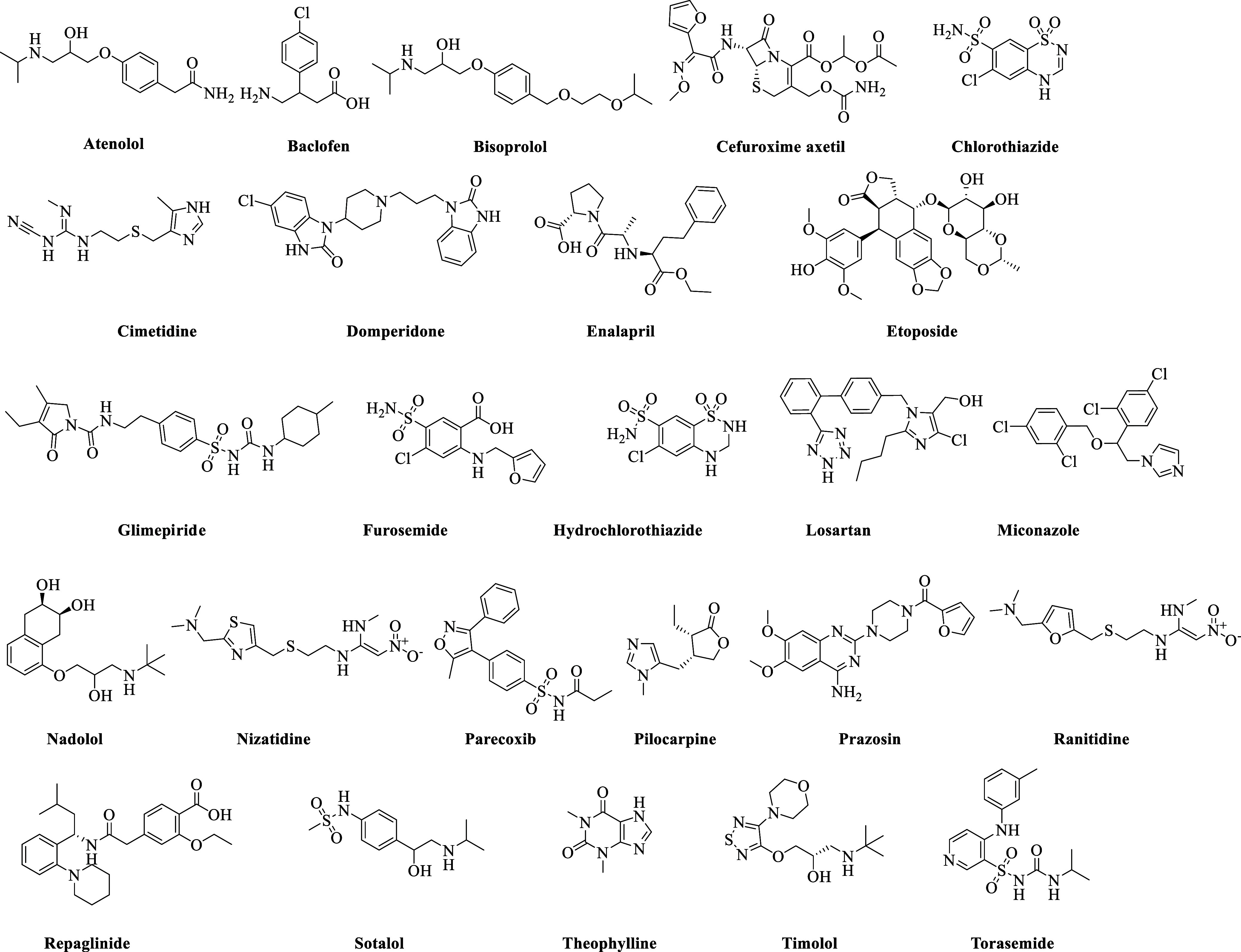
Chemical structures of CNS– standards used in the BBB permeability study.

Similarly, furosemide’s sulfonamide, amine, and carboxyl groups make it highly polar, which is a limiting factor in passive diffusion.^16,17,57^ In contrast, even though ibuprofen (*P*_m_ = 5.27) contains a carboxylic acid group (which is ionizable), its small molecular weight and predominantly hydrophobic aromatic ring enable sufficient lipophilicity.^16,17^ These observations suggest that when molecular weight is low and hydrophobic moieties predominate—as in compounds such as ibuprofen, caffeine, or propranolol—the detrimental effect of polar functional groups on CNS permeability can be attenuated.^16,17^

Interestingly, caffeine (*P*_m_ = 7.33) exhibits significantly greater blood–brain barrier permeability compared to theophylline (*P*_m_ = 1.05), likely due to its higher lipophilicity and reduced capacity for hydrogen bonding.^16,46^ The replacement of a hydrogen (N–H) in theophylline with a methyl (–CH_3_) in caffeine decreases hydrogen bond donation, making caffeine less polar and more membrane-permeable. Additionally, caffeine remains more neutral at physiological pH, reducing ionization-related permeability issues, whereas theophylline has more hydrogen bond donors, which restrict its passive diffusion across biological membranes. These differences explain why caffeine has stronger CNS effects, while theophylline primarily acts as a bronchodilator without BBB penetration. IAM chromatography’s ability to differentiate caffeine and theophylline, despite their single methyl group difference, highlights its effectiveness in mimicking biological membrane interactions. IAM chromatography can capture subtle changes in lipophilicity, hydrogen bonding, and thus membrane affinity, demonstrating its qualification to accurately predict passive diffusion in the case of BBB permeability.^39,40,48^

The CNS– category prominently features drugs with sulfonamide groups. Chlorothiazide (*P*_m_ = 0.05) and hydrochlorothiazide (*P*_m_ = 0.42) both incorporate sulfonamide or thiazide functional groups that introduce significant polarity into their structures. These groups form strong interactions with water molecules, thereby reducing the compound’s lipophilicity and its ability to traverse the BBB.^16,17^ Similarly, parecoxib (*P*_m_ = 0.39), with its bulky structure and sulfonamide moiety, is hindered by these polar characteristics.

Compounds like cimetidine (*P*_m_ = 0.86), which contains a guanidine group,^66^ and losartan with its ionizable tetrazole ring remain largely charged at physiological pH. The charge hinders their ability to cross the hydrophobic BBB because ionized compounds are less soluble in the lipid environment. This contrasts with CNS+ compounds that often avoid such strongly ionizable groups.^16,44^

In CNS+ drugs (Figure 4), the presence and management of ionizable groups are carefully optimized to enhance blood–brain barrier permeability by maintaining a balance between aqueous solubility and lipophilicity.^16,44^ Many contain weakly basic amines with p*K*_a_ values in the range between 8 and 10.5 (e.g., donepezil, rivastigmine, and clonidine), ensuring that a sufficient fraction of the molecules remains un-ionized at physiological pH to enable passive diffusion across the BBB. This minimizes their polarity, promoting an efficient diffusion across the BBB. Examples like clonidine (*P*_m_ = 5.25), donepezil (*P*_m_ = 2.20), and melatonin (*P*_m_ = 4.37) are either inherently weak or structured in a way that the overall molecule remains predominantly neutral at physiological pH due to their ionizable groups being either weak bases that are only partially protonated (clonidine, donepezil) or absent altogether (melatonin).^16,17,44^ In the case of clonidine and donepezil, the weakly basic nitrogens ensure that an un-ionized fraction is present, while their hydrophobic aromatic scaffolds reduce the polarity. In contrast, melatonin contains no strongly ionizable groups and remains entirely neutral under physiological conditions. This reduced level of ionization minimizes polarity and supports passive diffusion across the BBB.

**Figure 4:**
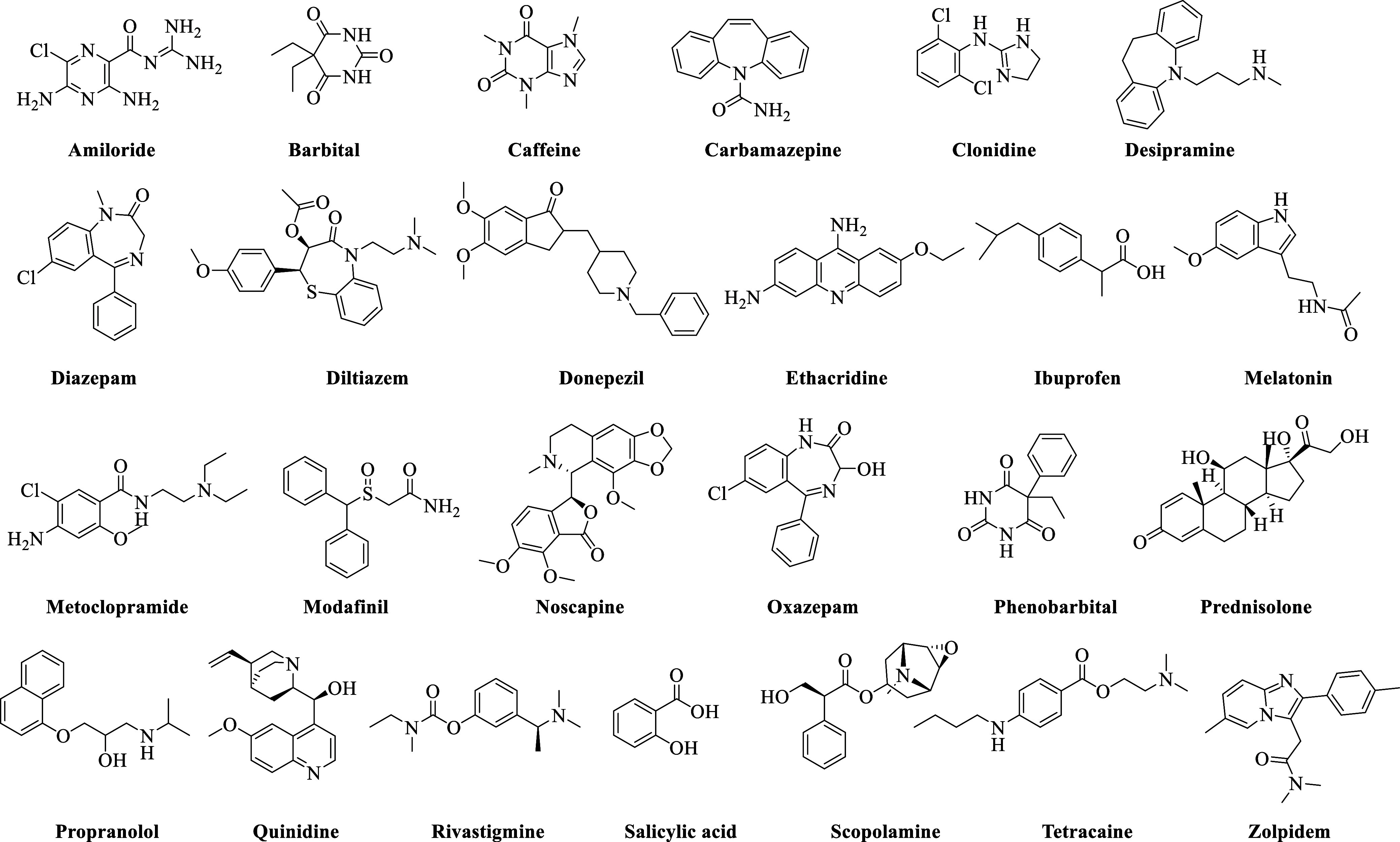
Chemical structures of CNS+ standards used in the BBB permeability study.

Many CNS+ compounds take advantage of planar aromatic structures to enhance the BBB permeability, e.g., carbamazepine (*P*_m_ = 10.76) and quinidine (*P*_m_ = 8.83) feature a planar, aromatic framework that minimizes polar interactions while promoting hydrophobic interactions with the lipid layer of the BBB. Diazepam (*P*_m_ = 7.03) and oxazepam (*P*_m_ = 7.29) similarly benefit from a benzodiazepine structure that is largely nonpolar, thereby facilitating passive diffusion.

Some drugs, such as metoclopramide (*P*_m_ = 1.51) and scopolamine (*P*_m_ = 1.57), fall near the threshold between CNS– and CNS+. Their structures reflect a delicate balance between polar and nonpolar groups. Metoclopramide contains one primary amine and one amide group and lipophilic regions (benzene and alkene moieties); small shifts in its structural makeup can tip its overall permeability. Scopolamine’s tropane alkaloid framework provides enough hydrophobic character to offset its polar features, resulting in borderline permeability. These examples underscore how minor structural modifications can have significant consequences on BBB permeability.

Molecular weight is a critical determinant of the BBB permeability. Generally, compounds with lower molecular weights are better suited for passive diffusion across the BBB.^16,44^ The importance of inclusion of molecular weight in the determination of P_m_ value is, for example, evident in comparison between atenolol (*k*_IAM_ = 0.310, MW = 266.3) and barbital (*k*_IAM_ = 0.318, MW = 184.2) or nizatidine (*k*_IAM_ = 0.299, MW = 331.5) and salicylic acid (*k*_IAM_ = 0.284, MW = 138.1). In these pairs, the IAM capacity factor is nearly identical, yet the lower molecular weight of barbital and salicylic acid plays a crucial role in determining *P*_m_, as smaller molecules face less steric hindrance during membrane permeation and, as such, have higher diffusion coefficients resulting in a higher *P*_m_. This illustrates how molecular size directly influences passive permeability, reinforcing the significance of the molecular weight in BBB penetration.

Maintaining a relatively low molecular weight ensures efficient penetration by minimizing steric hindrance and preserving a favorable balance between lipophilicity and aqueous solubility.^16,44^ Conversely, compounds with higher molecular weights often exhibit poorer permeability, as their larger size can hinder diffusion and is frequently accompanied by an increase in polar or ionizable groups that further limit BBB transport.

These findings support the implications for the rational design of CNS-active drugs. Optimizing drug candidates by targeting a molecular weight range of 150–350 Da combined with adequate lipophilicity is crucial for enhancing BBB permeability via passive diffusion.^16,44^

In addition, reducing excessive polarity—by limiting hydrogen bond donors, acceptors, and ionizable groups—can mitigate unfavorable interactions with the blood–brain barrier and support passive permeability.^16,44^ Incorporating planar, aromatic moieties further improves permeability, as demonstrated by compounds such as carbamazepine and quinidine. On the contrary, for drugs intended to remain peripherally active, strategies may involve deliberately increasing the molecular weight and/or polarity to prevent unwanted CNS penetration.

The limitation of this study lies in the fact that the permeability model derived is based on a passive diffusion framework, which assumes that transport across the blood–brain barrier is primarily governed by membrane partitioning and diffusion through the lipid bilayer. IAM chromatography mimics the interaction of compounds with phospholipid membranes and therefore captures the lipophilicity-driven component of BBB permeability. However, it does not account for transporter-mediated processes such as carrier-mediated uptake or active efflux by proteins including *P*-glycoprotein (*P*-gp), breast cancer resistance protein (BCRP), or other ATP-binding cassette transporters. Consequently, the present model should be interpreted as an estimator of passive permeability potential rather than a comprehensive predictor of overall brain exposure. For compounds whose BBB transport is strongly influenced by active transport mechanisms, deviations from the predicted permeability values may occur. Nevertheless, passive diffusion remains the dominant transport mechanism for many small, moderately lipophilic CNS-active drugs, making IAM chromatography a useful tool for early-stage permeability screening.

Another commonly used method in early drug discovery is the parallel artificial membrane permeability assay (PAMPA), which provides complementary information regarding drug transport across biological membranes.^67^ Several compounds included in the present IAM data set have also been evaluated in PAMPA-BBB assays. Comparison with literature data reveals consistent permeability trends between the two methods. Highly permeable compounds such as diazepam, propranolol, and carbamazepine exhibit high permeability in both IAM and PAMPA models, reflecting their favorable lipophilicity and low polarity.^67–69^ Conversely, hydrophilic drugs including atenolol, nadolol, and hydrochlorothiazide display low permeability in both systems due to their high polarity and limited ability to partition into lipid membranes.^67,68^ These observations indicate that both IAM chromatography and PAMPA-BBB assays capture similar qualitative permeability trends for highly lipophilic and highly polar compounds, even though the two approaches probe different physicochemical aspects of membrane transport.

IAM chromatography represents a simplified biomimetic system that primarily captures passive membrane-partitioning processes. While it cannot reproduce active transport mechanisms or the full cellular complexity of biological BBB models, its experimental simplicity, reproducibility, and compatibility with high-throughput screening make it a valuable early-stage tool for estimating passive permeability and guiding medicinal chemistry optimization prior to more resource-intensive *in vitro* and *in vivo* studies.^40,43,70^

### 3.2 QSAR Study on Intestinal Absorption Based on IAM Chromatography

The ability of a compound to cross biological membranes is largely determined by its physicochemical properties, such as polarity, lipophilicity, and molecular size, influencing the interaction with lipid membranes.^44,65,71^

In this QSAR study, a data set of 20 structurally diverse compounds (Table 5) was compiled to develop a predictive model using multiple linear regression (MLR), with each compound characterized with the chromatographic IAM retention factor and physicochemical predictors polar surface area (PSA), molecular weight (MW), molar volume (MV), and molar refractivity (MR). The resulting regression model is shown in eq 8:

8 $$\%HIA=\left(126.728\pm 18.334\right)-\left(0.658\pm 0.228\right)\times PSA+\left(0.842\pm 0.823\right)\times k_{IAM}+\left(0.460\pm 0.267\right)\times MW-\left(0.288\pm 0.238\right)\times MV-\left(0.952\pm 1.147\right)\times MR$$

for which *n* = 20, *R* = 0.839, *R* ^2^ = 0.704, *R* ^2^_adj_ = 0.599, *s* = 14.418, and *F* = 6.673.

**Table 5:** List of Compounds and Their Physical Properties used for QSAR Study^a^

compound	observed %HIA	PSA	logP	PSA × logP	1/*k*_IAM_ × logP	1/*k*_IAM_	*k*_IAM_ [*n* = 3] ± CV [ %]	molecular weight	molar volume	molar refractivity	surface tension
amiloride^42^	50	156.79	1.08	169.33	1.95	1.81	0.55 ± 0.70	229.6	108.5	49.9	112.5
benzylpenicillin^63^	30	112.01	1.67	187.06	4.85	2.91	0.34 ± 0.87	334.4	235.1	86.3	67.8
carbamazepin^42^	84	46.33	2.67	123.70	1.16	0.44	2.30 ± 0.48	236.3	186.5	69.7	57.3
cefuroxime^200^	44	199.06	0.47	93.56	0.35	1.33	1.33 ± 3.35	510.5	241.0	96.7	78.0
chlorothiazide^64^	70	135.44	–0.22	–29.80	–0.71	3.22	0.31 ± 1.81	295.7	144.4	61.8	96.5
desipramine^62^	100	15.27	4.13	63.07	0.29	0.07	14.16 ± 4.32	266.2	254.2	84.2	39.9
diazepam^62^	100	32.67	2.91	95.07	0.15	0.05	19.46 ± 0.31	284.7	225.8	809	46.1
enalapril^64^	60	95.94	2.43	233.13	3.52	1.45	0.69 ± 0.58	376.5	312.5	99.5	51.2
etoposide^64^	50	160.82	0.3	48.25	0.18	0.60	1.67 ± 0.64	588.2	378.5	140.1	76.4
furosemide^63^	61	131.01	3.10	406.13	2.58	0.83	1.20 ± 2.55	330.7	205.8	75.8	75.2
hydrochlorothiazide^63^	67	135.12	–0.07	–9.46	–0.02	0.22	4.53 ± 2.95	297.7	175.8	62.7	62.0
ibuprofen^64^	93	37.29	3.72	138.72	0.71	0.19	0.19 ± 3.54	206.0	200.3	60.8	38.0
indometacin^64^	100	68.53	3.1	212.44	0.37	0.12	8.65 ± 1.76	357.0	269.5	94.6	47.4
minoxidil^3^	90	93.62	–0.41	–38.38	–0.60	1.45	0.69 ± 0.49	209.3	137.6	54.6	71.5
nadolol^64^	32	81.95	1.29	105.72	6.30	4.88	0.21 ± 1.49	309.4	260.0	85.7	46.6
paracetamol^64^	80	49.33	0.34	16.77	1.96	5.75	1.74 ± 1.10	151.1	120.9	42.4	52.8
propranolol^64^	93	41.49	3.1	128.62	0.62	0.20	4.97 ± 0.09	259.3	237.1	79.0	42.6
quinidine^64^	80	45.59	3.44	156.83	1.55	0.45	2.20 ± 0.46	324.4	266.3	95.8	56.0
ranitidine^64^	55	111.56	1.23	137.22	3.12	2.54	0.39 ± 0.85	350.9	265.4	85.6	45.0
scopolamine^63^	90	62.30	0.76	47.35	0.83	1.09	0.92 ± 2.25	303.4	230.9	80.4	56.3

a Values in the Observed %HIA column are literature values for the human intestinal absorption. The physicochemical parameters PSA, logP, MW, MV, MR, and ST were calculated with Biovia Draw. *k*_IAM_ was measured in triplicate.

The negative coefficient for PSA (−0.658) indicates that a higher polar surface area decreases absorption, consistent with reduced membrane permeability of highly polar compounds. The coefficient for *k*_IAM_ is positive (0.842), suggesting that greater lipophilicity (higher retention on the IAM HPLC column) improves absorption. Although the overall model has a moderately good adjusted coefficient of correlation (*R* ^2^ = 0.599), the individual contributions of MW, MV, and MR are statistically nonsignificant (*p*-value >0.1). This suggests that the mentioned molecular descriptors do not contribute meaningfully to the prediction of the %HIA and only add unnecessary complexity to the model. The initial regression model incorporated all these variables, and the improved models take into consideration their interaction terms (e.g., PSA·logP and 1/*k*_IAM_ * logP).

Recognizing the irrelevance of the descriptors MW, MV, and MR in eq 8, the model shown in eq 9 is simplified by retaining only PSA and *k*_IAM_, descriptors that are statistically significant:

9 $$\%HIA=\left(91.118\pm 9.447\right)-\left(0.263\pm 0.080\right)\times PSA+\left(1.265\pm 0.788\right)\times k_{IAM}$$

for which *n* = 20, *R* = 0.768, *R* ^2^ = 0.589, *R* ^2^_adj_ = 0.541, *s* = 15.420, and *F* = 12.205. Although the adjusted coefficient of determination falls to 0.541 from 0.599, this model benefits from improved statistical robustness for the two predictors, as the *F* value rises from 6.673 to 12.205. Together, these results highlight PSA as the dominant negative predictor and *k*_IAM_ as the primary positive predictor of %HIA. The removal of the nonsignificant variables minimizes the overfitting of the model and focuses on the variables that are most influential.

In eq 10, the *k*_IAM_ variable is transformed into its reciprocal (1/*k*_IAM_), which captures a nonlinear relationship with %HIA more effectively:

10 $$\%HIA=\left(107.189\pm 6.658\right)-\left(0.305\pm 0.061\right)\times PSA-\left(5.557\pm 1.907\right)\times \left({1/k}_{IAM}\right)$$

for which *n* = 20, *R* = 0.827, *R* ^2^ = 0.685, *R* ^2^_adj_ = 0.648, *s* = 13.513, and *F* = 18.462. This transformation improves the model’s explanatory power, as seen by the increase in adjusted *R* ^2^ from 0.589 to 0.648 and a reduction in the standard error (s) from 15.420 to 13.513. The negative coefficient for 1/*k*_IAM_ indicates that higher *k*_IAM_ values (which result in a lower reciprocal) are associated with increased %HIA.

Building on the previous model, eq 11 incorporates logP as an additional physicochemical predictor to capture lipophilic effects of the compounds:

11 $$\%HIA=\left(135.204\pm 12.194\right)-\left(0.429\pm 0.071\right)\times PSA-\left(7.353\pm 2.822\right)\times logP-\left(8.257\pm 1.946\right)\times \left({1/k}_{IAM}\right)$$

for which *n* = 20, *R* = 0.882, *R* ^2^ = 0.779, *R* ^2^_adj_ = 0.737, *s* = 11.672, and *F* = 18.761.

The introduction of this variable not only increases the adjusted *R* ^2^ to 0.737 from 0.648 but also further reduces the standard error to 11.672, indicating a better fit. The negative effect of the coefficient for logP suggests that an increase in lipophilicity is associated with a decrease in %HIA, as excessive lipophilicity can reduce aqueous solubility or promote membrane binding, which lowers absorption.

Equation 12 marks a significant improvement by incorporating an interaction term between 1/*k*_IAM_ and logP:

12 $$\%HIA=\left(108.086\pm 5.201\right)-\left(0.297\pm 0.037\right)\times PSA+\left(1.132\pm 1.285\right)\times logP-\left(8.053\pm 0.787\right)\times \left({1/k}_{IAM}\cdot logP\right)$$

for which *n* = 20, *R* = 0.968, *R* ^2^ = 0.938, *R* ^2^_adj_ = 0.926, *s* = 6.195, and *F* = 80.187.

The introduction of this interaction captures the synergistic effect of steric hindrance (1/*k*_IAM_) and lipophilicity (via logP).

Equation 13 represents the most refined model (Figure 5) by incorporating both the interaction between PSA and logP and the interaction between 1/*k*_IAM_ and logP:

13 $$\%HIA=\left(122.366\pm 4.527\right)-\left(0.417\pm 0.035\right)\times PSA-\left(5.269\pm 1.584\right)\times logP+\left(0.092\pm 0.019\right)\times \left(PSA\cdot logP\right)-\left(9.882\pm 0.641\right)\times \left({1/k}_{IAM}\cdot logP\right)$$

for which *n* = 20, *R* = 0.988, *R* ^2^ = 0.975, *R* ^2^_adj_ = 0.969, *s* = 4.038, and *F* = 147.221. This comprehensive model achieves an adjusted coefficient of determination of 0.969, indicating that the high variability in %HIA is explained by the predictors. The standard error decreases to 4.038, indicating that the average prediction error is within ∼4% HIA. The model’s robustness is further supported by the *F* value of 147.221, which is more than an order of magnitude higher than earlier models (e.g., *F* = 12.205 in eq 9). The coefficients also provide clear mechanistic insights: the PSA has a strong negative effect (−0.417 ± 0.035), logP also contributes negatively (−5.269 ± 1.584), while the interaction PSA·logP has a small but statistically significant positive contribution (+0.092 ± 0.019), indicating that the impact of polarity depends on lipophilicity. The introduced interaction between the IAM and logP (1/*k*_IAM_·logP) has a large negative coefficient (−9.882 ± 0.641), showing that highly lipophilic compounds with low IAM retention are strongly penalized in absorption. The extremely low standard error and high *F* statistics further prove the robustness and predictive strength of this final model.

**Figure 5:**
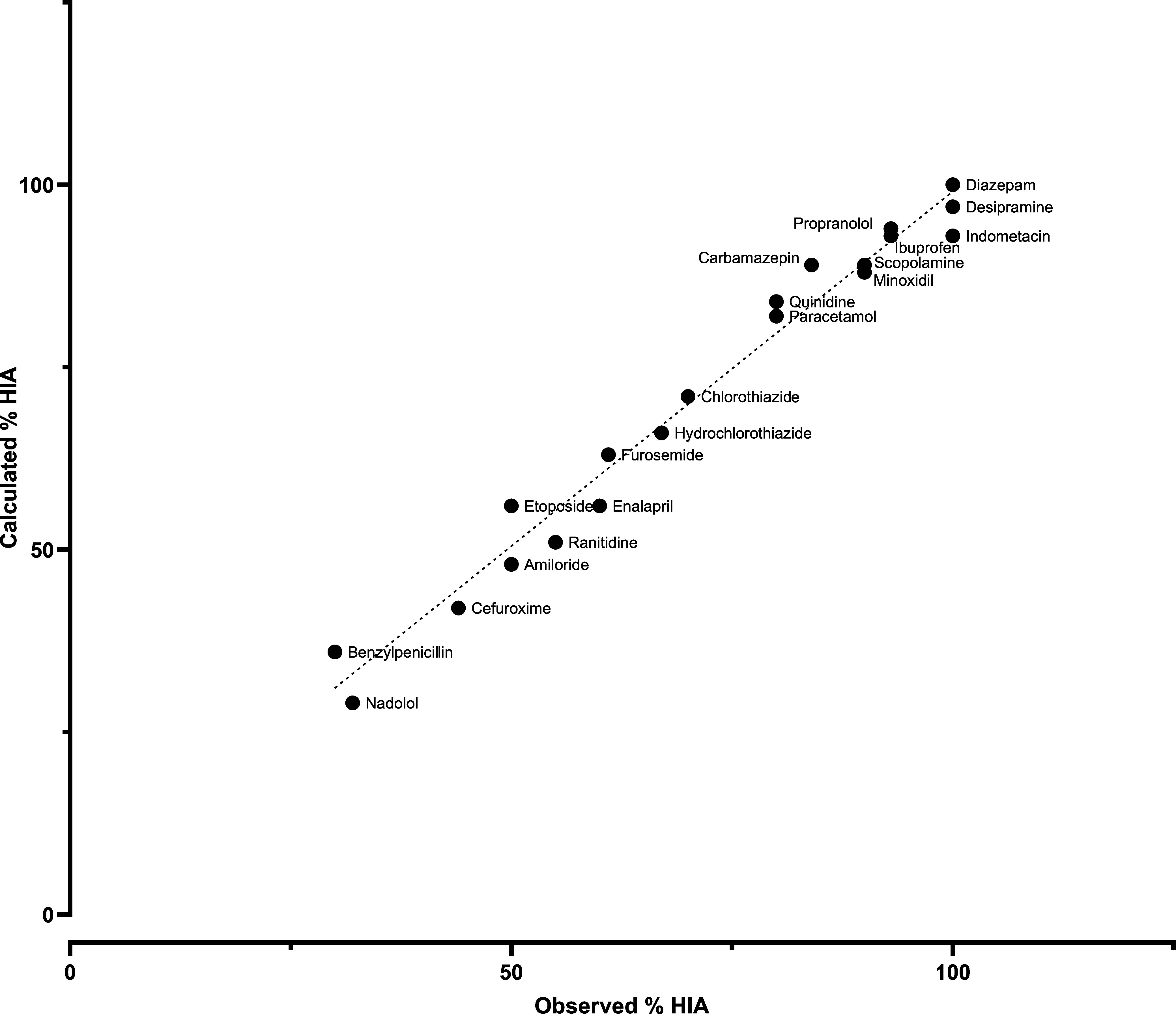
QSAR validation plot: comparison between calculated and predicted %HIA.

IAM chromatography mimics the interaction of molecules with biological membranes, in particular, the phosphatidylcholine-rich lipid bilayers of enterocyte cell membranes in the intestinal epithelium. The strong negative coefficient for the interaction term between 1/*k*_IAM_ and logP underscores the importance of experimental retention data in predicting the intestinal absorption.

PSA is a well-established descriptor of a molecule’s hydrogen bonding capacity and overall polarity. In this model, PSA exhibited a strong negative coefficient, suggesting that compounds with higher PSA values tend to have reduced intestinal absorption. This is consistent with the concept that a high polar surface area (due to the presence of polar moieties, e.g., hydroxyl, amino, or carboxyl groups) increases PSA and may reduce absorption due to increasing the likelihood of hydrogen bonding, which can hinder passive diffusion across the lipid-rich membranes of the intestinal epithelium.^65,71^

This influence of PSA in QSAR modeling of intestinal absorption of drugs aligns with previous studies, indicating that compounds with PSA values above 150 Å^2^ exhibit poor passive intestinal absorption, while those below 90 Å^2^ tend to be well absorbed in general.^44,71^ This trend is evident in our data set, where poorly absorbed compounds such as cefuroxime (PSA = 199.1 Å^2^, %HIA = 44%) and etoposide (PSA = 160.82 Å^2^, %HIA = 50%) show limited intestinal uptake. In contrast, highly absorbed drugs like Diazepam (PSA = 32.7 Å^2^, %HIA = 100%) and Ibuprofen (PSA = 37.3 Å^2^, %HIA = 93%) demonstrate efficient passive diffusion. The underlying mechanism is that higher PSA values lead to increased hydrogen bonding capacity, which reduces lipophilicity and hinders membrane permeability. This is consistent with Lipinski’s Rule of Five, which suggests that compounds with a PSA value higher than 150 Å^2^ are less likely to be orally bioavailable.^44^

The only two structurally similar compounds used in this model, chlorothiazide (calculated %HIA = 71%) and hydrochlorothiazide (calculated %HIA = 66%), confirm the significance of the data delivered from IAM chromatography. Hydrochlorothiazide differs from chlorothiazide by saturation of the 3,4-double bond, adding two extra hydrogen atoms to the ring. The difference in the interaction with the lipid-like stationary phase of the biomimetic column results in hydrochlorothiazide’s smaller 1/IAM value (0.22) in comparison to chlorothiazide (3.22). Combined with the logP and PSA descriptors, it reflects a small increase in polarity, which slightly reduces its ability to partition into lipid membranes of the epithelium. Ionization also plays a role in membrane interactions, as evidenced by the differences in k_IAM_ values at varying pH levels. At pH 5.5, chlorothiazide exhibits a *k*_IAM_ of 0.31, while hydrochlorothiazide reaches 4.53. In contrast, at pH 7.4, the *k*_IAM_ values decrease to 0.04 for chlorothiazide and 0.33 for hydrochlorothiazide. These shifts highlight the impact of ionization state on retention behavior. This fact underlines how relatively small structural changes, such as the addition of hydrophilic moieties in hydrochlorothiazide compared to chlorothiazide, can impact the absorption of drugs by altering their membrane affinity and permeability.

The logP value serves as a measure of a compound’s lipophilicity, a critical factor for membrane permeation. logP value plays a dual role in intestinal absorption, balancing membrane permeability and aqueous solubility. In general, compounds with logP values between 1.5 and 5 tend to exhibit optimal intestinal permeability, whereas those with logP <1.5 were found to be too hydrophilic, and those with logP 5 are too lipophilic to be efficiently absorbed.^31^ This trend is reflected in the data set, where, e.g., highly absorbed compounds such as ibuprofen (logP = 3.72, calculated %HIA = 93%) and propranolol (logP = 3.10, calculated %HIA = 94%) fall within the optimal logP range. In contrast, nadolol (logP = 1.29, calculated %HIA = 29%) and cefuroxime (logP = 0.47, calculated %HIA = 42%) demonstrate reduced intestinal uptake due to the hydrophilic nature of the compounds. Although this model shows a marginally significant coefficient (*p* < 0.005) for logP in comparison to other descriptors of the model (*p* < 0.001) when considered alone, its inclusion is essential because lipophilicity influences both solubility and the ability to partition into the lipid bilayer of the epithelium. A balanced logP value is crucial, as excessive lipophilicity (due to, e.g., alkyl or aromatic groups) may lead to poor aqueous solubility and rapid metabolism, while insufficient lipophilicity may prevent effective epithelial membrane penetration. The significant positive interaction between PSA and logP in this model indicates that the negative impact of high PSA on absorption can be partially mitigated when a compound possesses a certain level of lipophilicity. This suggests that for compounds with moderately high PSA, increased lipophilicity might enhance their ability to traverse the membrane by favoring partitioning into the lipid phase, thereby offsetting some of the adverse effects of polarity.^65,71^

## 4 Conclusion

This study highlights the effectiveness of IAM chromatography as a predictive tool for evaluating passive permeability across the blood–brain barrier and intestinal absorption. The strong correlation between IAM retention factors (*k*_IAM_) and permeability confirms the ability of this biomimetic chromatographic approach to mimic drug–membrane interactions, making it a valuable, fast, and cheap alternative to more complex *in vitro* and *in vivo* models, also allowing for high-throughput screening.

The BBB permeability model established in this study effectively combines k_IAM_ and molecular weight to predict passive diffusion across the blood–brain barrier. The strong correlation between k_IAM_ and membrane permeability supports the role of IAM chromatography as a biomimetic tool for evaluating drug–membrane interactions. Since IAM retention reflects the partitioning of compounds into phospholipid-like environments, it serves as a useful experimental surrogate for passive transport across the BBB. However, molecular weight also plays a crucial role, as smaller molecules (<400–450 Da) generally exhibit better BBB penetration, while larger molecules seem to face steric and diffusional limitations. The study confirms that while high k_IAM_ values are associated with increased BBB permeability, molecular weight acts as a limiting factor, particularly for compounds exceeding the optimal range of 150–350 Da. This is evident in cases where two compounds with similar *k*_IAM_ values but different MWs exhibit varying permeability, emphasizing the inverse relationship between size and diffusion efficiency.

The developed intestinal absorption model, combining IAM chromatography with *in silico* molecular descriptors, demonstrates a powerful approach for predicting the drug permeability. IAM chromatography, which mimics phospholipid membrane interactions, captures essential properties like lipophilicity and membrane affinity, while in silico descriptors such as PSA and logP provide additional mechanistic insights into hydrogen bonding and solubility. The strong predictive power of the final model (*R* ^2^_adj_ = 0.969) confirms that IAM retention data significantly enhance absorption predictions beyond traditional molecular descriptors alone. The inclusion of interaction terms (e.g., 1/kIAM × logP) further highlights the complex interplay between experimental retention behavior and molecular properties, refining the accuracy of permeability assessments. This integrative approach not only improves oral bioavailability predictions but also streamlines early drug discovery, offering a cost-effective and biomimetic alternative to traditional permeability assays that are ready for high-throughput screening.

Overall, IAM chromatography proves to be a rapid, cost-effective, and physiologically relevant method for early-stage drug screening, particularly for CNS-active compounds and orally administered drugs. By integration of IAM retention data with computational approaches, drug discovery efforts can better predict permeability, optimize molecular properties, and enhance the selection of promising candidates for further development. These findings support the broader application of IAM chromatography in ADME profiling, contributing to more efficient drug design and improved pharmacokinetic prediction.

## §

The Florey Institute of Neuroscience and Mental Health, 30 Royal Parade, Parkville, VIC 3052, Australia
